# Migratory Thrombophlebitis and Acute Q Fever

**DOI:** 10.3201/eid1003.030859

**Published:** 2004-03

**Authors:** Manuel L. Fernandez Guerrero, Pablo Rivas, Rosa Garcia Delgado

**Affiliations:** * Fundacion Jimenez Diaz, Madrid, Spain

**Keywords:** Q fever, migratory thrombophlebitis, Trousseau syndrome

**To the Editor**: Q fever is a worldwide zoonosis particularly common in some cattle- raising countries such as Great Britain, France, Spain, Australia, and Canada (*1*). Widespread use of diagnostic serologic tests has led to increasing recognition of the disease in many other parts of the world. For example, Q fever has been recently described in Southeast Asia (*2*).

Acute Q fever usually manifests as a febrile syndrome with hepatic abnormalities or pneumonia; chronic Q fever is commonly associated with endocarditis (*3*,*4*). When the disease manifests with an atypical clinical picture, diagnosis becomes difficult.

We describe a patient in whom Trousseau’s syndrome developed as the sole manifestation of acute Q fever. To the best of our knowledge, this complication has not been found previously in patients with Q fever.

A 39-year-old, previously healthy man was admitted to the hospital because of fever and subcutaneous painful induration in the legs and arms. Ten days previously, he had fever, generalized aches, and malaise. Forty-eight hours after the onset of these symptoms, he noticed a painful induration and redness along the right calf that spontaneously subsided in a few days. He remained febrile, and additional swollen, painful indurated lesions appeared on his thighs and arms. Pleuritic chest pain developed, and he was taken to the emergency room.

On admission, he was febrile (38.7°C). A tender, indurated cord, 10 cm long, was felt on the greater saphenous vein of the right thigh. The cephalic vein of the left arm and the basilic vein of the right arm were also tender and indurated.

The leukocyte count was 9,800/mm^3^; hemoglobin 14.5 g/dL, and platelets 441,000/ mm^3^. The fibrinogen levels (385 mg/dL) and prothrombin time (12 s, control 10–14 s) were normal. The partial thromboplastin time (40 s, control 26–36 s) was mildly prolonged. Serum aspartate aminotransferase was 75 μ/L, serum alanine aminotransferase was 110 μL, and γ-glutamyltranspeptidase was 230 μ/L. Antibodies to phospholipids (aPL) measured by enzyme-linked immunosorbent assay were positive (titer of 95 for antibodies of class immunoglobulin (Ig)/titer of 19 for antibodies of class IgM. Results of testing for tumor-associated antigens, such as carcinoembryonic antigen, carcinoma antigen 19-9, prostatic specific antigen, and alpha fetoprotein, were negative. The x-ray films of the chest and computed tomography scan of the thorax and abdomen were normal. The patient was treated with subcutaneous heparin and diclophenac, and fever and migratory thrombophlebitis subsided.

Because the patient had been working with manure several days before his initial symptoms, Q fever serologic testing was requested. The antibody levels measured by complement fixation (CF) against phase II *Coxiella burnetii* antigen was 1:512. By indirect immunofluorescence, the titers of IgM and IgG against phase I and II were 1:64 and 1:512 and 1:256 and >2,048, respectively. Antibody titers against *Mycoplasma, Chlamydia, Legionella*, enterovirus, and influenza were negative.

Recovery was uneventful and the patient was asymptomatic during a follow-up visit 3 weeks later. Antiphospholipid antibodies were negative. Three months after the acute phase of the infection, new titers of antibodies (CF) against *C. burnetii* were 1:128. Two years after the episode the patient was asymptomatic.

This patient is unique in that he had acute Q fever with migratory thrombophlebitis. A diagnosis of Trousseau’s syndrome associated with an occult malignancy was considered on admission, but it was excluded soon. The recent history of exposure to manure was the key for the clinical diagnosis. Although specific anti-coxiella treatment was not given, the patient followed a self-limited course, and both clinical and laboratory abnormalities promptly subsided.

Microscopic vasculitis and thrombosis are commonly found in patients with other rickettsial infections (*5*), but vascular phenomena must be considered an exceptional event in patients with Q fever. However, thrombophlebitis and pulmonary embolisms have been occasionally reported (*6*–*8*). These unusual manifestations have been associated with aPL during the course of acute Q fever (*7*,*8*).

Antibodies to phospholipids have been found in 80% of patients in a large series of acute Q fever (*9*). None of the patients in the study showed thrombotic events or cardiac valve involvement in contrast to patients with lupus or primary aPL syndrome in whom clinical manifestations attributed to aPL developed (*9*). This observation could be explained by the fact that aPL found in patients with lupus and primary aPL syndrome are directed against a complex antigen that includes a lipid-binding inhibitor of coagulation, which has been characterized as a β2-glycoprotein I (apolipoprotein H). This glycoprotein seems to inhibit the activation of the contact phase system of the intrinsic pathway of blood coagulation (*10*). On the other hand, apolipoprotein H is not necessary for the aPL activity observed in patients with Q fever and other infectious diseases (*10*).

According to these studies, the observation of low titers of aPL in the serum of our patient during the acute phase of Q fever must be seen as a finding of uncertain importance not necessarily associated with migratory thrombophlebitis. In short, migratory thrombophlebitis (Trousseau’s syndrome) should be added to the ever- growing list of unusual manifestations of Q fever.
